# Navigating Latency-Inducing Viral Infections: Therapeutic Targeting and Nanoparticle Utilization

**DOI:** 10.34133/bmr.0078

**Published:** 2024-10-16

**Authors:** Arathy Vasukutty, Yeonwoo Jang, Dongwan Han, Hansoo Park, In-Kyu Park

**Affiliations:** ^1^ Department of Biomedical Sciences and BioMedical Sciences Graduate Program (BMSGP), Chonnam National University Medical School, Jeollanam-do 58128, Republic of Korea.; ^2^ School of Integrative Engineering, Chung-Ang University, Seoul 06974, Republic of Korea.

## Abstract

The investigation into viral latency illuminates its pivotal role in the survival strategies of diverse viruses, including herpesviruses, HIV, and HPV. This underscores the delicate balance between dormancy and the potential for reactivation. The study explores the intricate mechanisms governing viral latency, encompassing episomal and proviral forms, and their integration with the host’s genetic material. This integration provides resilience against cellular defenses, substantially impacting the host–pathogen dynamic, especially in the context of HIV, with implications for clinical outcomes. Addressing the challenge of eradicating latent reservoirs, this review underscores the potential of epigenetic and genetic interventions. It highlights the use of innovative nanocarriers like nanoparticles and liposomes for delivering latency-reversing agents. The precision in delivery, capacity to navigate biological barriers, and sustained drug release by these nanocarriers present a promising strategy to enhance therapeutic efficacy. The review further explores nanotechnology's integration in combating latent viral infections, leveraging nanoparticle-based platforms for drug delivery, gene editing, and vaccination. Advances in lipid-based nanocarriers, polymeric nanoparticles, and inorganic nanoparticles are discussed, illustrating their potential for targeted, efficient, and multifunctional antiviral therapy. By merging a deep understanding of viral latency’s molecular underpinnings with nanotechnology’s transformative capabilities, this review underscores the promise of novel therapeutic interventions. These interventions have great potential for managing persistent viral infections, heralding a new era in the fight against diseases such as neuroHIV/AIDS, herpes, and HPV.

## Introduction

Latency-inducing viral infections represent a fascinating aspect of virology, where viruses persist within a host in a dormant state, evading detection and remaining asymptomatic [1]. This phase, prevalent in virus families like herpesviruses and human immunodeficiency virus (HIV), signifies a pivotal strategy for viral survival [2]. The 2 primary forms of viral latency, episomal and proviral, delineate distinct mechanisms by which viruses persist within host cells [3]. Episomal latency sees viral genes stabilized as separate entities in the host cell’s cytoplasm or nucleus, while proviral latency involves integration of the viral genome into the host’s genetic material [4]. Each form of latency bears its own susceptibility to degradation, with proviral latency offering more resilience against cellular mechanisms aiming to eradicate viral genetic material. The implications of viral latency extend far beyond simple dormancy. They play a fundamental role in the host–pathogen interplay, allowing viruses to persist despite the host’s immune responses and adverse environmental conditions. Moreover, the reversibility inherent in latency permits these viruses to reactivate, producing viral progeny when certain environmental cues prompt their resurgence. These latent infections can endure for prolonged periods, requiring mechanisms for sustained infection maintenance [5]. Understanding these intricate processes, particularly evident in viruses like HIV, offers insights into the clinical manifestations of infections and unveils potential avenues for managing persistent viral diseases.

The rarity of true latency among viruses underscores the uniqueness of this phenomenon, with HIV serving as primary examples. The distinctive attributes of HIV latency stem from the infrequency of latently infected cells, the complexities associated with investigating and discerning these cells, the multifaceted nature of latency, and the intricacies involved in comprehending and addressing the latent reservoir in HIV infection [6–8]. Therapeutic strategies tackling viral latency, particularly concerning herpesviruses and HIV, have been under extensive investigation [9]. Long-term administration of nucleoside analogs like valaciclovir or famciclovir has demonstrated success in suppressing recurrences of lesions in individuals harboring latent herpesviruses [10]. However, the eradication of latent reservoirs, notably in HIV, remains a formidable challenge. Exploration of epigenetic and genetic factors governing virus latency suggests a potential target for therapeutic intervention [11]. Despite incremental advancements, addressing viral latency persists as a crucial yet elusive challenge in managing persistent viral infections, highlighting the ongoing complexity in devising treatment strategies for these conditions.

**Fig. 1.**
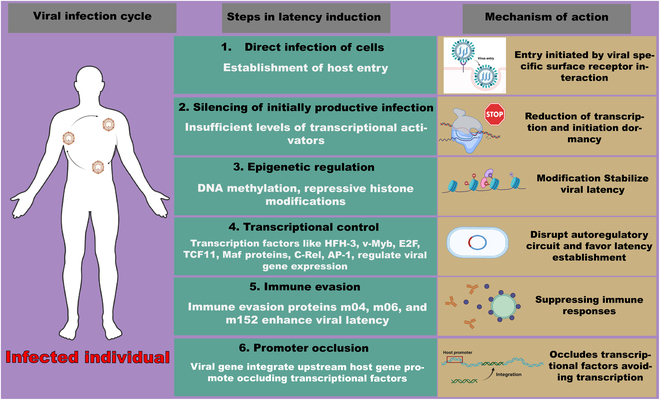
Summary of general molecular mechanisms involved in latency induction. (1) Direct infection occurs when an individual encounters a virus, which then attaches to the host cell surface and enters through specific viral entry surface proteins unique to each virus. (2) Inadequate transcriptional activators result in the reduction and eventual dormancy of the virus. (3) DNA methylation and histone modifications lead to epigenetic alterations, aiding in stabilizing the viral genome integrated into host cells. (4) Viral-specific transcriptional factors such as HFH-3, v-Myb, E2F, TCF11, Maf, C-Rel, and AP-1 disrupt host transcription regulators, promoting latency. (5) Immune evasion proteins, secreted from infected host cells, help to suppress the immune response. (6) Viral genes integrated upstream of host genes assist in circumventing transcription processes. Created in BioRender.

As shown in Fig. 1, the molecular steps involved in inducing latency in viruses are complex, involving a series of processes that allow the virus to establish a dormant state within the host cell. After viral entry, latency can occur when productive viral infections in activated cells are silenced during their transition to a resting memory state due to insufficient transcription. Epigenetic mechanisms, such as DNA methylation, histone modifications like H3K9me3 and H3K27me3, and noncoding RNAs, play a crucial role in silencing viral chromatin and limiting viral transcription during latency by stabilizing latency. This is especially evident in herpesviruses such as herpes simplex virus (HSV) and Epstein–Barr virus (EBV) [3]. Also, the presence of repressors like viral-specific transcriptional factors such as HFH-3, v-Myb, E2F, TCF11, Maf, C-Rel, and AP-1 disrupts host transcription regulators, contributing to latency. Viral proteins, noncoding RNAs, and immunosuppressive cytokines help evade immune responses and create a cellular environment favorable for latency, with cell type-specific restrictions, like inefficient viral promoter activation in neurons, facilitating latency, particularly in HSV infections [12].

Epigenetic modifications are pivotal in viral survival, enabling viruses to manipulate host gene expression and evade immune responses by altering histone modifications, DNA methylation patterns, and noncoding RNA expression [13]. These mechanisms create an environment conducive to viral replication and persistence, facilitating immune evasion, viral latency, and the regulation of both viral and host gene expression. Insights into these processes have led to potential therapeutic applications, including the development of epigenetic drugs such as histone deacetylase (HDAC) inhibitors, targeting viral epigenetic modifiers, and employing epigenetic editing techniques [14]. These interventions aim to reactivate latent viruses, restore host antiviral gene expression, and reverse virus-induced epigenetic changes [15]. Additionally, understanding epigenetic regulation can inform vaccine design and adjuvant selection, potentially enhancing immune responses to vaccination [16].

Building on these insights, therapies targeting epigenetic modifications offer promising approaches for treating viral latency. Key strategies include using HDAC inhibitors, DNA methyltransferase (DNMT) inhibitors, and histone methyltransferase inhibitors to reactivate latent viruses [17]. Bromodomain inhibitors and targeted epigenetic editing using clustered regularly interspaced short palindromic repeats (CRISPR)-based technologies are also being explored. By disrupting the epigenetic silencing of latent viruses, these approaches make viruses vulnerable to elimination through antivirals or immune responses [18]. To enhance efficacy, combination therapies and modulation of cellular metabolic pathways are being investigated. Despite their potential, challenges remain in achieving specificity, minimizing off-target effects, and ensuring complete viral eradication. Ongoing research focuses on optimizing these approaches for clinical application in treating persistent viral infections.

To overcome these challenges, nanocarriers such as nanoparticles and liposomes have emerged as promising tools for delivering inhibitors in a site-specific and targeted manner. These carriers boast several advantages in delivering latency-reversing agents and antiviral drugs. One key advantage lies in their targeted delivery capability, engineered to precisely reach specific cell types, including viral reservoirs [19]. This precision could potentially elevate drug concentrations at infection sites, bolstering drug efficacy while minimizing unintended effects elsewhere [20–22]. Additionally, nanocarriers enable sustained drug release, crucial for providing prolonged exposure of latent viral reservoirs to latency-reversing agents, overcoming challenges posed by the short half-lives of certain drugs and supporting the reversal of viral latency. Moreover, their small size grants nanocarriers enhanced penetration abilities through biological barriers, including those surrounding viral reservoirs [23]. This increased penetration potential significantly improves drug delivery to latent infection sites, potentially augmenting latency reversal. Importantly, by minimizing exposure of healthy tissues to high drug concentrations, nanocarriers hold the promise of reducing systemic toxicity, a vital consideration in therapy development for persistent viral infections [24]. Furthermore, their capacity for co-delivering multiple drugs offers a pathway for combination therapy, targeting various facets of viral latency simultaneously and potentially heightening intervention efficacy against the intricate mechanisms associated with viral persistence. While the potential of nanocarriers in reversing viral latency is promising, their clinical application necessitates further investigation and validation. However, their capacity to enhance the effectiveness of latency-reversing strategies for HIV, herpes, human papillomavirus (HPV), and other persistent viral infections remains a subject of burgeoning interest, paving the way for potential advancements in antiviral therapy. In this comprehensive review, our emphasis has been directed toward elucidating the molecular mechanisms that contribute to latency in viral infections, as well as exploring the potential of nanoparticles at each stage of this process.

## Epigenetic Modifications for Virus Survival and Their Therapeutic Applications

Epigenetic alterations play a crucial role in the establishment and persistence of viral latency in various infections, including HIV, herpesviruses, HPV. These modifications significantly contribute to the regulation of viral gene expression and the maintenance of the latent state [25,26]. These modifications profoundly impact the latency of HIV, with research emphasizing the role of histone modifications and DNA methylation [27]. Human herpesviruses employ cellular epigenetic mechanisms to ensure reactivation and progeny production, illustrating the significance of epigenetic regulation in establishing latency. In HPV infections, the viral genome undergoes epigenetic silencing during initial infection, with alterations in these modifications tied to the host cell’s differentiation status. Studies reveal epigenetic heterogeneity in HIV-1 latency establishment, emphasizing the intricate interplay between epigenetic regulation and viral latency establishment [28]. Moreover, the well-characterized epigenetic repression of herpesviruses upon infection contributes to latency establishment, while HPV demonstrates altered epigenetic regulators and reprogramming in infected cells. General mechanisms involved with epigenetic modifications are histone modifications, DNA methylation, chromatin remodeling, and noncoding RNAs. We have compiled a comprehensive overview of the primary epigenetic modifications associated with prevalent viral infections, presented in Table 1.

**Table 1.** Comprehensive overview of major epigenetic modifications related to viruses, along with therapeutic strategies targeting these modifications

Sl no.	Virus	Epigenetic modifications	Therapeutic targeting approach	Ref.
1.	HIV (human immunodeficiency virus)	Epigenetic reprogramming for controlling viral gene expression or latency	“Shock and kill” strategy to switch on latent HIV provirus into an active state, and “block and lock” strategy to permanently silence the virus in latent reservoirs	[11]
2.	HSV (herpes simplex virus)	Interplay between histone modifications, DNA methylation, and noncoding RNAs in promoting viral latency and reactivation	Inhibitors of histone deacetylases, demethylases, acetyltransferases, and methyltransferases, as well as inhibitors of DNA methyltransferases have shown efficiency in repressing viral infections or reactivating latency	[29]
3.	EBV (Epstein–Barr virus)	Selective elimination of EBV genomes from latently infected cells and inhibition of viral reactivation from latency	“Shock and kill” strategy using drugs to activate latent viruses, and “block and lock” strategy to permanently silence the virus in latent reservoirs	[30]
3.	HCMV (human cytomegalovirus)	Recruitment of host chromatin-modifying enzymes for transcriptional silencing of integrated provirus	Reactivating HCMV from the latent phase (“shock and kill” paradigm) or repressing the virus lytic to control the infection and its complications	[31]

### Histone modifications

Histone modifications wield significant influence in establishing and sustaining viral latency in HIV and herpes infections. In HIV-1, the provirus undergoes silencing orchestrated by host chromatin-modifying enzymes that govern histone modifications, a process reversible by transcriptional control mechanisms [29]. Posttranslational histone modifications crucially impact HIV’s lytic infection, reinforcing latency and facilitating reactivation. The interplay between histone acetyltransferases (HATs) and HDACs significantly regulates histone modifications, affecting the accessibility of HIV-1 long terminal repeats (LTR) to transcription factors [30].

**Fig. 2.**
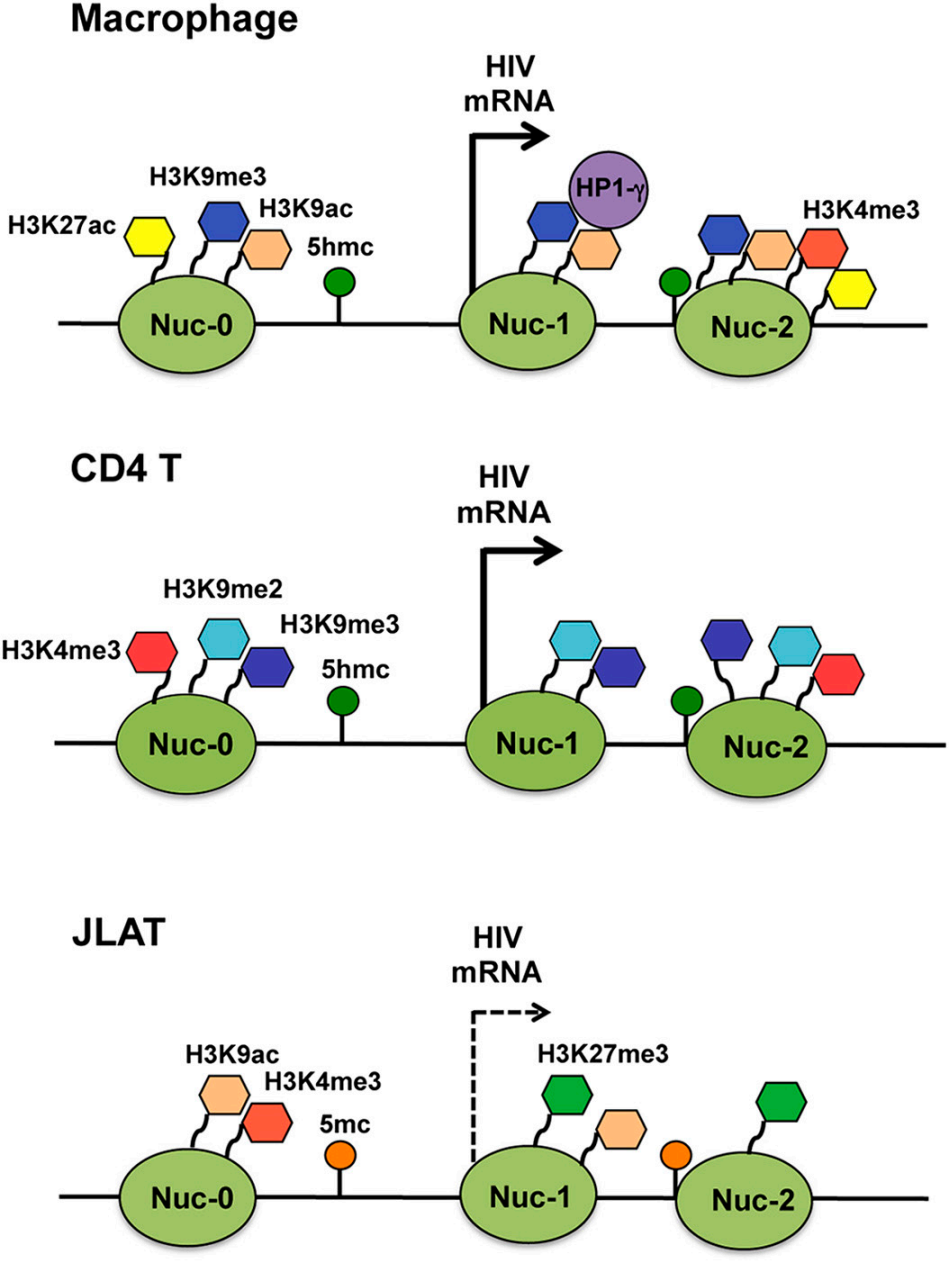
This scientific presentation outlines the model of HIV epigenetic regulation, providing a condensed overview of histone tail modifications linked to the LTR region in HIV-infected MDM, CD4 T cells, and JLAT 8.4 cells. It highlights the unique bivalent chromatin structure in MDMs, characterized by high levels of H3K9me3 and H3K27ac, compared to the different histone modification patterns observed in CD4 T cells and JLAT cells. Image reprinted from [35], 2022 American Society for Microbiology.

Two key repressive histone methylation marks are H3K27me3 and H3K9me3. The deposition of H3K27me3 by the polycomb repressive complex 2 (PRC2) is linked to facultative heterochromatin, promoting chromatin compaction and thus aiding in the transcriptional silencing of viral genes [31,32]. This modification has been observed in latent HSV and EBV genomes. H3K9me3, catalyzed by histone methyltransferases such as SUV39H1, is linked to constitutive heterochromatin and recruits heterochromatin protein 1 (HP1), leading to chromatin condensation. It is found on latent HIV provirus and herpesvirus genomes [30,33,34]. Research conducted by Lu et al. [35] revealed that in macrophages (MDMs), HIV sequences exhibit enrichment of H3K9me3 throughout the viral genome. This enrichment is characterized by an unusual bivalent histone modification, featuring high levels of both H3K9me3 and H3K27ac. Their research also shows that 5′-hydroxymethylcytosine (5hmC) is abundant throughout the HIV genome in MDMs and CD4 cells, unlike JLAT cells, which display higher levels of 5mC (Fig. 2). These results indicate unique epigenetic regulatory features in MDMs compared to both productively infected CD4 T cells and latently infected JLAT cells, suggesting variations that might differ from previously recognized macrophage activation states.

Histone deacetylation also contributes to viral latency. HDACs remove acetyl groups from histones, resulting in a more compact chromatin structure that restricts access to transcription factors. HDACs are recruited to viral promoters during latency establishment, and deacetylation of histones H3 and H4 is associated with silencing of HIV and herpesvirus genomes. These modifications often collaborate to establish and maintain viral latency. For example, H3K9me3 can recruit HDACs, thereby reinforcing transcriptional repression. The balance between activating marks (e.g., acetylation) and repressive marks (e.g., methylation) determines the transcriptional state of the viral genome [36].

### DNA methylation

DNA methylation emerges as a crucial player in establishing and perpetuating viral latency in HIV and herpes infections [37]. In HIV latency, DNA methylation profiles in infected individuals correlate with disease progression and antiretroviral response, indicative of its influence on gene expression and associated cascades. Hypermethylation of the 5′-LTR in HIV proviral DNA potentially curtails viral replication, contributing to latency maintenance [38]. Moreover, the interplay between histone and DNA methylation in silencing latent HIV proviruses generates intricate and varied patterns of modifications, reinforcing viral latency through epigenetic mechanisms (Fig. 2) [39,40]. In herpes infections like HSV, chromatin assembly on viral DNA, coupled with DNA methylation and histone modifications, orchestrates the suppression of viral gene expression during latency [41].

These findings underscore the significance of DNA methylation in orchestrating viral latency in HIV and herpes infections. Understanding the dynamics of DNA methylation and its impact on viral gene expression holds pivotal importance in devising targeted therapeutic interventions aimed at reversing viral latency [42]. Targeting DNMT activity emerges as a strategy to impede or reverse the DNA methylation of viral genomes, aiming to reactivate latent viruses [43]. DNMT inhibitors (DNMTis) such as azacytidine and decitabine have demonstrated potential in reactivating latent HIV by demethylating the viral promoter. These nucleoside analogs, already Food and Drug Administration (FDA)-approved for other indications, possess well-characterized pharmacology but encounter challenges related to specificity and potential toxicity [44]. Nonnucleoside DNMTis like RG108 and SGI-1027 may offer more targeted inhibition with fewer side effects, although their efficacy in viral reactivation requires further validation [45]. Combination approaches, such as combining DNMTi with HDAC inhibitors, have shown synergistic effects in some studies [46]. Emerging technologies such as CRISPR-dCas9 fused to DNA demethylases hold promise for locus-specific demethylation of viral promoters, potentially offering precise targeting with reduced off-target effects [47]. However, significant challenges persist in achieving specificity, managing toxicity, ensuring complete and sustained viral reactivation, and addressing the heterogeneity of latent reservoirs. The intricate nature of epigenetic silencing mechanisms suggests that targeting DNA methylation alone may be insufficient. Therefore, combination therapies with other latency-reversing agents and immunotherapies are warranted.

### Chromatin remodeling proteins

**Fig. 3.**
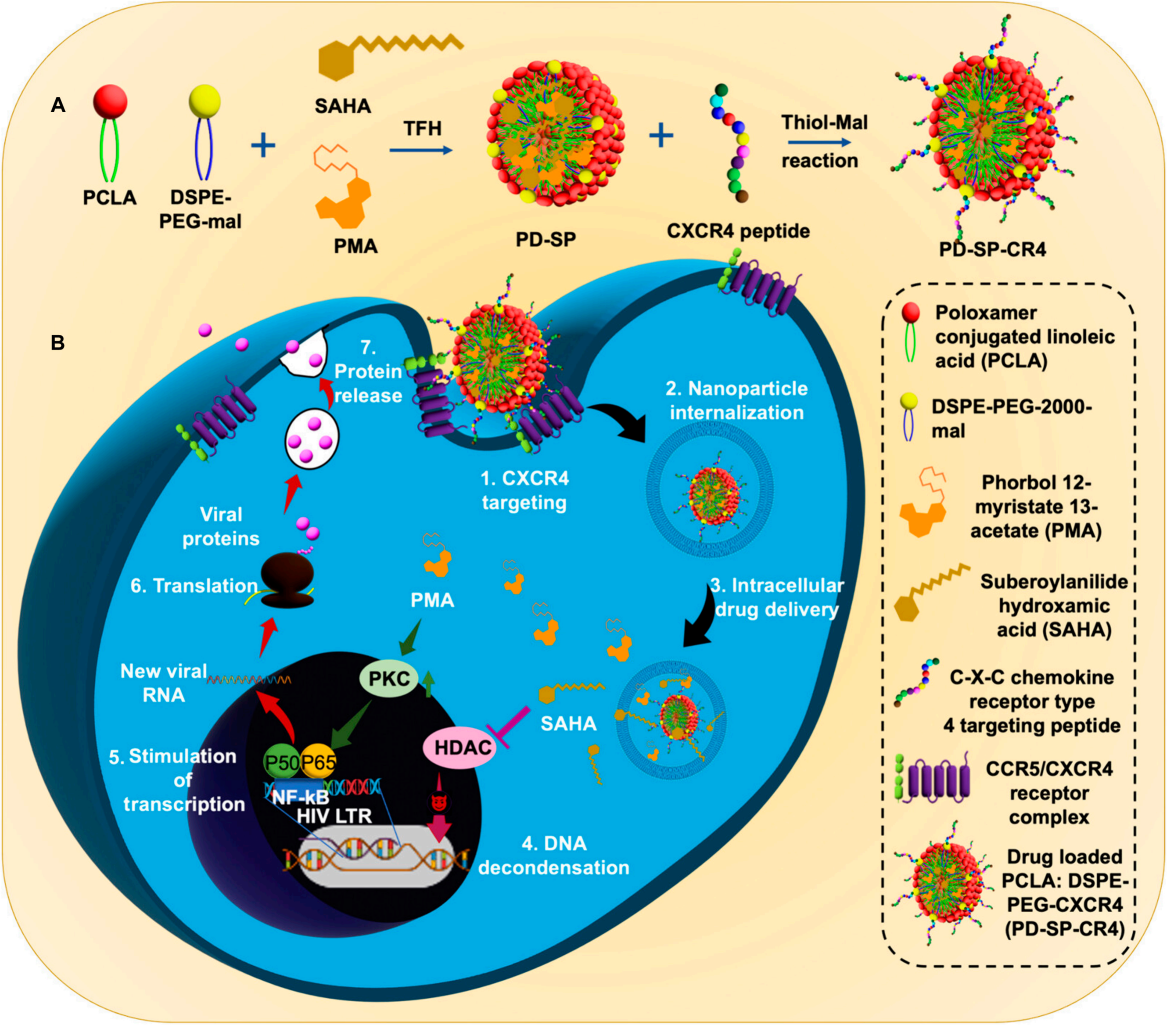
Therapeutic approach to overcome HIV-1 latency by combined delivery of HDACi and PKC using polymeric nanoparticle. Image reprinted from [52], 2023 American Chemical Society.

Chromatin remodeling proteins emerge as pivotal players in shaping and sustaining viral latency across infections like HIV, herpes, papilloma, and cytomegalovirus (CMV) [48,49]. In HIV-1 infection, proteins like HATs contribute to restructuring the chromatin landscape within the viral LTR region, instrumental in establishing viral latency. Additionally, the HDAC family of chromatin remodeling proteins plays a substantive role in perpetuating HIV latency. In HSV infections, chromatin assembly on viral DNA and histone modifications, alongside transcriptional regulation of the latency-associated transcript (LAT), orchestrates a suppression of viral gene expression, reinforcing latency [50,51]. Our research team was engaged in formulating nanoparticles for the combined delivery of HDACi and protein kinase C (PKC) to address chromatin remodeling, aimed at reversing HIV latency (Fig. 3) [52]. Similarly, human CMV (HCMV) gene repression during latency occurs through the heterochromatinization of viral genomes, underscoring the significance of chromatin remodeling in latency maintenance [53].

The interplay between HATs and HDACs is crucial in regulating the accessibility of the HIV-1 LTRs to transcription factors, significantly impacting viral latency and reactivation. HATs add acetyl groups to histone tails, neutralizing their positive charge and weakening histone–DNA interactions, resulting in a more open chromatin structure that facilitates easier access for transcription factors [54]. Conversely, HDACs remove these acetyl groups, restoring the positive charge and strengthening histone–DNA interactions, leading to a more compact chromatin structure that restricts transcription factor access [55]. In latently infected cells, HDACs predominantly occupy the HIV-1 LTR, maintaining a repressive chromatin environment that contributes to viral latency [56]. However, upon cellular stimulation, HATs can be recruited to the LTR, acetylating histones and creating an open chromatin structure. This allows for the binding of key transcription factors such as nuclear factor κB (NF-κB) and recruitment of the viral Tat protein, which further enhances HAT activity and recruits P-TEFb. This cascade promotes efficient viral transcription and reactivation.

These discoveries highlight the crucial involvement of chromatin remodeling proteins in regulating viral latency in a range of infections, including HIV, herpes, papilloma, and CMV. Grasping the intricacies of chromatin remodeling dynamics and their influence on viral gene expression is of utmost significance in developing precise therapeutic approaches targeted at reversing viral latency. Mastering these mechanisms holds the potential for significant advancements in interrupting latency, introducing innovative strategies for the management of enduring viral infections.

### Noncoding RNAs

**Fig. 4.**
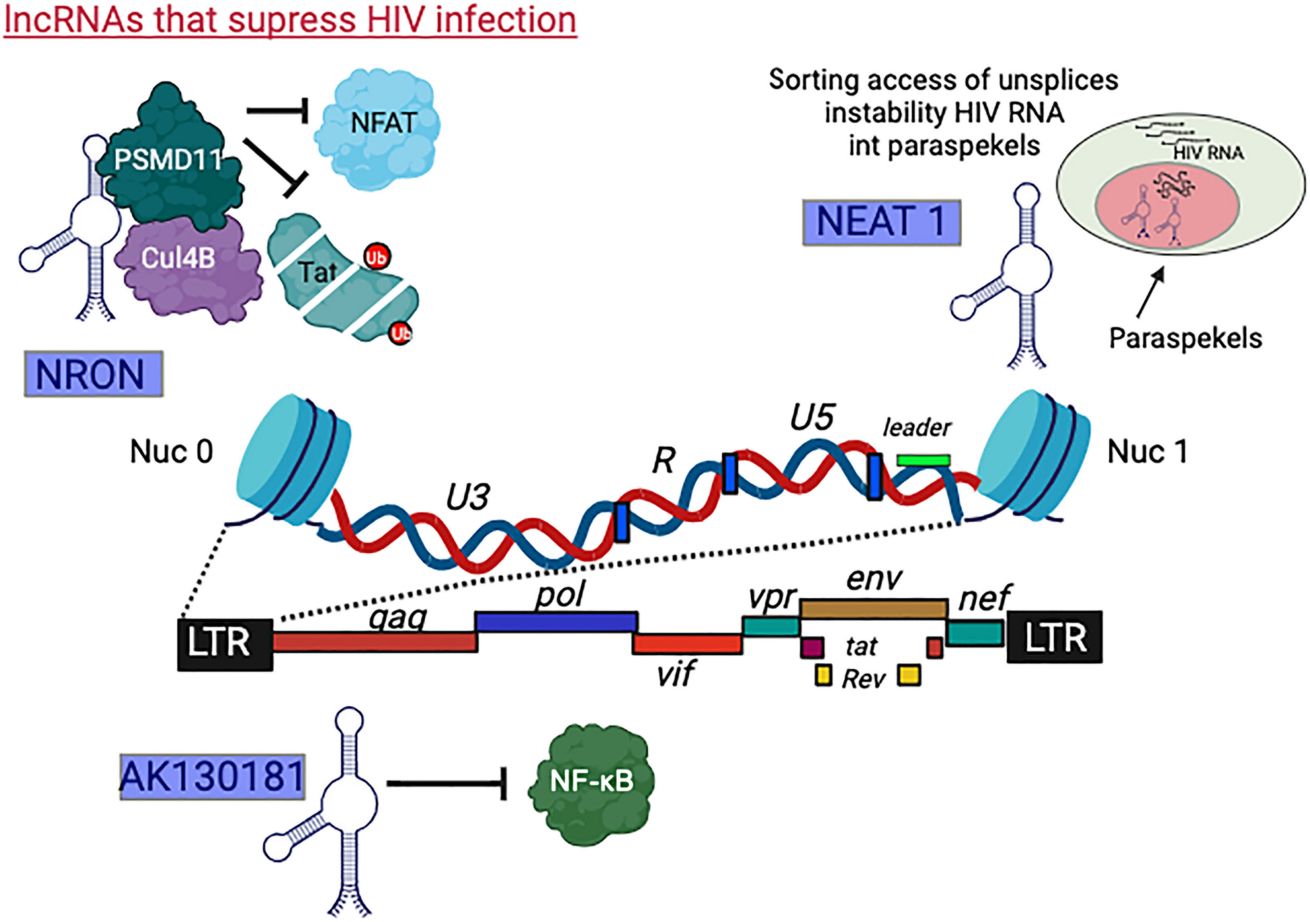
This scientific discourse focuses on long noncoding RNAs (lncRNAs) that function as suppressors of HIV gene expression. A specific subset of noncoding RNAs inhibits HIV infection through diverse molecular mechanisms. NRON impedes HIV-1 gene expression in primary CD4^+^ T cells by preventing NFAT's nuclear translocation, while NEAT1 causes HIV transcripts to colocalize to paraspeckles as unspliced transcripts. Additionally, AK130181 suppresses NF-κB-dependent activation of the HIV promoter, with its silencing promoting HIV activation in infected primary CD4^+^ T resting cells. Image reprinted from [127] under the terms of the Creative Commons Attribution 4.0 International license 2024.

Noncoding RNAs, including long noncoding RNAs (lncRNAs), microRNAs (miRNAs), and small noncoding RNAs (sRNAs), are implicated in regulating viral latency across infections like HIV, herpes, papilloma, and CMV. In HIV latency, the HIV-1 antisense protein (ASP) RNA recruits the PRC2 to the HIV-1 promoter, inducing repressive chromatin formation [57,58]. miRNAs also contribute to HIV latency by targeting the HIV-1 LTR, maintaining viral latency. In HSV infections, the LAT from HSV-1 prevents apoptosis and suppresses lytic chromatin, favoring facultative heterochromatin on lytic promoters. Furthermore, sRNA1 and sRNA2 expression during HSV-1 latency, along with latent miRNAs, repress lytic gene transcription and impact viral replication. In HPV infections, lncRNAs influence viral gene expression, exhibiting changes in expression within HPV-infected cells. CMV latency involves lncRNAs RNA2.7 and RNA4.9, with lnc4.9 binding to PRC2 and suppressing lytic gene expression. These insights underscore the diverse roles of noncoding RNAs in shaping viral latency mechanisms, offering a deeper understanding of their involvement across different infections and presenting potential targets for therapeutic interventions aimed at manipulating viral latency. It has been shown that a subset of lncRNAs inhibits HIV infection through various molecular mechanisms (Fig. 4) [48]. NRON inhibits the NFAT transcription factor, thus preventing HIV-1 gene expression in primary CD4^+^ T cells by obstructing NFAT's entry into the nucleus. NEAT1, an essential part of paraspeckles, acts as a scaffold for these nuclear structures. During a viral infection, elevated NEAT1 levels result in HIV transcripts localizing to paraspeckles as unspliced transcripts. Furthermore, AK130181, which is highly expressed in resting CD4^+^ T cells, inhibits the activation of the HIV promoter by NF-κB. Its role in maintaining latency is evident as HIV activation increases when AK130181 is silenced in infected primary resting CD4^+^ T cells from patients.

## Emerging Therapeutic Strategies Using Nanoparticle

**Fig. 5.**
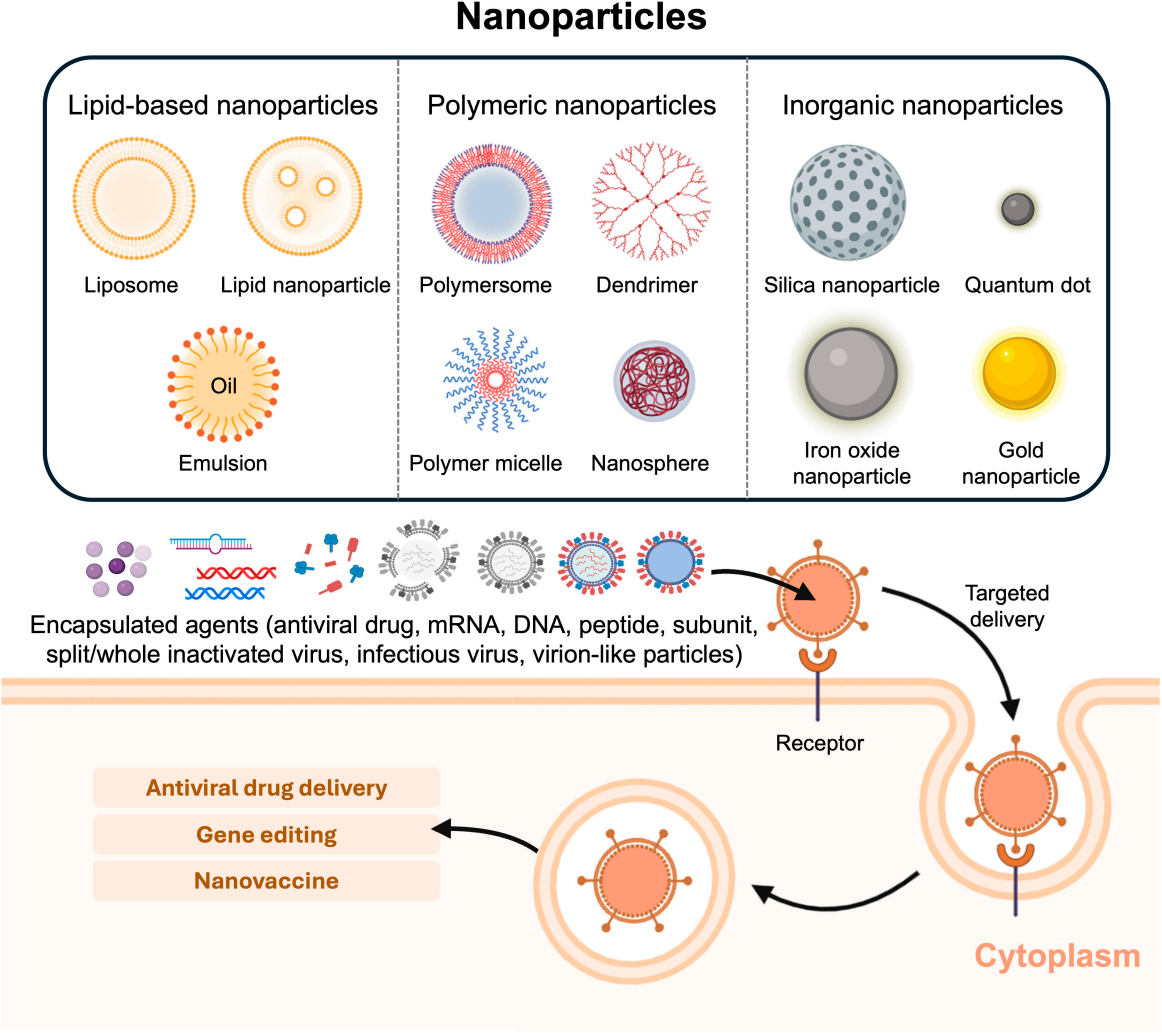
Overview of nanoparticle types and biomedical applications in antiviral therapy. Nanoparticles are categorized into lipid-based, polymeric, and inorganic types based on their structures and compositions. Various therapeutic agents, including antiviral drugs, mRNA, DNA, and peptides, can be encapsulated within these nanoparticles to enhance targeted drug delivery, facilitate gene editing, and develop vaccines, thereby improving antiviral efficacy.

Nanotechnology is a new interdisciplinary area revolutionizing medicine in the 21st century. Several nanotechnology-based strategies for latency virus disease treatment have evolved in recent decades, achieving advanced progress in both treatment and prevention [59]. The strategies also have the potential to revolutionize latent infection disease therapies by nanoparticle-based strategies. Many researchers have utilized nanoparticle-based latency reversal drug delivery nanoplatform, nanoparticle-mediated gene editing against latency-inducing viruses, and nanoparticle-based vaccines (Fig. 5). Nanoparticles can be designed with various materials (e.g., lipid, polymer, and inorganic compound) for various biomedical applications [60–62]. These nanoscaled particles confer excellent advantages such as passive targeting [i.e., enhanced permeability and retention (EPR) effect], large surface areas, incorporation of various agents, and easy functionalization [63].

Antiviral drug-loaded nanoparticles have been developed as a promising drug delivery nanoplatform for latent infection disease treatment. Nanotechnology-based drug delivery nanoplatforms offer various benefits, such as the possibility of controlling antiviral drug release for longer circulation time in a blood vessel, effectively loading antiviral drugs into nanoparticles, delivering poorly water-soluble antiviral drugs, targeting antiviral drugs to virus-infected cells of latent reservoirs, and delivering of these drugs intracellularly [59,64]. The antiviral drug-loaded nanoparticles decrease the severity of acute infections and reduce viral reactivation frequency [65,66]. All these features indicate that nanoscale drug delivery platform would offer the most promising treatment method against latency virus in the clinic.

Gene-editing methods, such as CRISPR/CRISPR-associated protein 9 (Cas9) (CRISPR/Cas9) RNA-guided system, zinc finger nuclease (ZFN), homing endonuclease (HE; i.e., meganuclease), transcription activator-like effector nuclease (TALEN), or similar enzymes, represent a novel and promising approach against viral replication and infection by destroying or modifying the genetic material of latency viruses [67–71]. Gene-editing techniques have gained considerable attention as potential antiviral therapies since they enable the permanent disruption of specific genes, offering the possibility of sustained viral inhibition with few therapeutic interventions. Recent advances in understanding the interactions between DNA and transcription factors, DNA repair mechanisms, and bacterial defense mechanisms have made precise gene editing within cells a realistic approach. These technologies can be tailored to recognize specific DNA target sequences, facilitating site-specific gene editing [72]. Nanoparticle-mediated gene editing directly targets latent genomes for disrupting or eliminating latent viruses inducing recurrent diseases while preserving neurons [73]. Nanotechnology-based gene-editing nanoplatforms are effective delivery gene-editing agents to targeted cells because the nanoparticles can incorporate various moieties, such as chemical conjugates, proteins, and beads. Therefore, nanoparticle-mediated gene-editing strategies are less prone to immunogenicity [74].

Nanoparticle-based vaccines (nanovaccines) offered several advantages over conventional vaccines for addressing the challenges associated with retroviral infections [75]. By enhancing antigen stability, reducing probability of immune evasion from the virus, enabling targeted delivery to antigen-presenting cells (APCs), and boosting overall immunogenicity, nanovaccines had the potential to overcome the limitations of traditional vaccine approaches. One key benefit of nanovaccines was their ability to improve antigen stability and decrease probability of immune evasion. By encapsulating viral antigens with nanoparticles, nanovaccine effectively shield them from degradation by enzymes and environmental factors. This ensured that the antigens remained stable and intact until reaching their target cells, thereby enhancing the vaccine's efficacy and reducing the risk of immune evasion [76,77]. Moreover, nanovaccines were engineered to target specific cells, such as dendritic cells and macrophages. These cells are pivotal for initiating and modulating immune responses. By delivering antigens directly to these APCs, nanovaccines ensured more efficient immune system priming. This targeted delivery was particularly critical for retroviral vaccines, countering immune evasion tactics employed by these viruses, such as interference with APC function or induction of immune exhaustion. Additionally, nanovaccines enhanced immunogenicity by incorporating adjuvants within the nanoparticle formulation, eliciting a stronger and more specific immune response. The controlled release of antigens from nanoparticles mimicked natural infection, providing prolonged exposure and a robust immune response. Therefore, nanovaccines prevent the antigenic materials from quick disintegration and induction of a localized immune response [78,79], directly deliver antigens to APCs such as dendritic cells by moiety functionalization, and conserve antigens against proteolytic enzymes to improve immune system exposure [76,80,81].

Nanoparticles loaded with antiviral drugs, such as HDAC inhibitors, chromatin remodeling proteins, and noncoding RNAs, to induce histone modifications, DNA methylation, and chromatin remodeling for antiviral treatment have garnered significant interest in combating viruses. For example, poly-(lactide-co-glycolic acid) (PLGA)–polyethylene glycol (PEG) nanoparticles loaded with HDAC inhibitors selectively deliver antiviral drugs to targeted cells for HIV-1 treatment. These nanoparticles effectively decrease and inhibit the activation of latent viruses by modulating histones [82]. Additionally, for the treatment of HIV, oxygen-containing liposome encapsulating the HDAC inhibitor drug vorinostat enhances the effect of chemotherapy by containing oxygen and co-delivering drugs such as protein kinase A activators. By conjugating with CXCR4-binding peptides, the liposomes specifically target CD4^+^ T cells to efficiently deliver therapeutic agents [83]. Exosome nanoparticles containing RNAs encoding zinc finger protein fused with the active domain of DNA methyltransferase 3A target the HIV promoter, inducing inhibition of virus expression. In vivo results demonstrate that through methyltransferase (ZPAMt), HIV expression is reduced by enhancing HIV DNA methylation suppression [84]. Topoisomerase IIβ (Topo IIβ), which regulates gene expression through chromatin remodeling in nonproliferating and differentiated cells, is expressed in HIV and is known to be inhibited by Topo II inhibitors, blocking DNA synthesis and virus replication. Protein nanoparticles deliver Topo IIβ-specific siRNA to suppress the expression of Topo IIβ and restrict chromatin remodeling, thereby reducing HIV replication [85]. Viral transcription inhibitors act as regulators of the viral promoter through short noncoding RNAs and chromatin remodeling factors [86]. miRNA, capable of modulating gene expression at the posttranscriptional level, is encapsulated in dendrimers to inhibit the replication of HIV. These dendrimers enhance biocompatibility by consisting of 4 different carbosilane dendrimers. Dendrimer-encapsulated miRNA is effective in preventing HIV replication by protecting and delivering miRNA to cells [87].

In this section, we introduce various delivery system (e.g., lipid-based nanocarrier, polymeric nanocarrier, and inorganic nanocarrier), which are used for a drug delivery nanoplatform, gene editing, and nanovaccine for disease treatment from latency viruses such as HIV, EBV, CMV, HPV, HSV, and VZV.

### Lipid-based nanoparticle

The relentless pursuit of more effective and targeted antiviral therapies has led to the innovative use of nanotechnology, specifically through the development of nanocarriers for drug, gene editing, and nanovaccine delivery. Lipid-based nanocarriers have emerged as powerful tools in the fight against viral infections (Table 2). Lipid-based nanocarriers are considered a promising antiviral agent delivery system because they can load either hydrophilic or hydrophobic agents and protect them from gastrointestinal degradation and various metabolic processes [24]. Furthermore, lipid-based nanocarriers show many advantages including modifications of agent pharmacokinetics, control of tissue distribution patterns, low immunogenicity, and high biocompatibility [88].

**Table 2.** Example of lipid-based nanoparticles for antiviral therapy. This table summarizes various lipid-based nanoparticles, encapsulated agents they carry, their target viruses, and key properties.

Lipid-based nanoparticle				
Nanoparticle	Encapsulated agents	Targeted virus	Properties of nanoparticles	Ref.
Folate-decorated liposome	Rilpivirine	HIV	Antiviral drug delivery Targeting ability to virus-infected macrophages	[92]
HLA-DR antibody-decorated liposome	Amphotericin B (AmB)	HIV	Antiviral drug delivery Targeting HLA-DR-expressed cells, which play a key role in HIV reservoirs	[93]
Membrane-derived nanoparticles	Cas9-sgRNA complexes	T cell-related virus	Gene-editing agent delivery Targeting virus-infected T cells The generation of chimeric antigen receptor T cells through genome editing	[94]
Lipid-based nanovaccines presenting HIV-1 viral proteins	BG505.SOSIP.664 (SOSIP)	HIV	Nanovaccine efficient uptake by dendritic cells (DCs) Humoral immunity against HIV-1	[95]
Liposome	Interferon α-2b (IFNα-2b)	HPV	Antiviral drug delivery Mucus-penetrating ability	[97]
Flexible nanoliposomes	siRNA targeting recombinant open reading frame 7 (r/si-ORF7)	VZV	Gene-editing agent delivery Delivery of antigen to in cellular setting and 3D human epidermal skin model	[98]
Immune-evasive liposome	Enfuvirtide (EFV), protoporphyrin IX (PPIX)	HIV	Antiviral drug delivery Immune-evasive ability Lipophilic drug loading	[99]

Especially, lipid-based nanocarriers with surface-attached antibodies can target the virus-infected mononuclear macrophages, which serve as viral reservoirs. For example, liposomes were decorated with folate to target macrophages overexpressing folate receptors. The folate-decorated liposome loading rilpivirine efficiently targeted activated macrophages, leading to effective antiviral drug delivery to HIV-infected macrophages for anti-HIV treatment [89]. Liposomes decorated with anti-HLA-DR (human leukocyte antigen-DR isotype) antibodies also showed specific binding to heterodimeric transmembrane glycoprotein HLA-DR-expressed cells, which play a key role in HIV reservoirs, since the virion surface physically presents the HLA-DR determinant [90]. The amphotericin B (AmB)-loaded liposomes decorated with anti-HLA-DR antibodies exhibited high inhibition of HIV-1 replication, whereas there was no significant antiviral activity from free AmB [90]. Antibody fragments presented on the surface of membrane-derived nanoparticles, which encapsulate the CRISPR/Cas9 protein and its guide RNA, enable the precise delivery of genome editing tools to target cells by recognizing specific cell surface markers. For example, Cas9-sgRNA complex, formed by the Cas9 endonuclease and a single guide RNA (sgRNA), packaging enveloped delivery vehicles (Cas9-EDVs), modified with scFv (single-chain variable fragment) targeting molecules that can target mutated form of the vesicular stomatitis virus G protein (VSVGmut), offer a more targeted approach to genome editing compared to traditional vectors, utilizing specific antibody–antigen interactions to deliver the genome editing tools directly to desired cells [91]. By employing multiple targeting molecules aimed at human T cells, Cas9-EDVs facilitate the creation of genome-edited chimeric antigen receptor T cells in humanized mice, effectively avoiding unintended delivery to nontarget cells such as liver hepatocytes [91]. Lipid-based nanovaccines designed to transport protein antigens and adjuvants enhance targeting to APCs, thereby elevating the immunogenic response beyond what is achieved with traditional soluble antigen proteins. For example, lipid-based nanovaccines loaded with BG505.SOSIP.664 (SOSIP) and presenting HIV-1 viral proteins have demonstrated efficient uptake by dendritic cells (DCs) in vitro, attributed to the preservation of crucial conformational epitopes, which fosters DC maturation and antigen presentation [92]. Rabbits immunized with these SOSIP-enriched lipid-based nanovaccines generated potent antigen-specific antibody responses, showcasing significant humoral immunity against HIV-1 [92]. Therefore, liposomes decorated with ligands attaching to virus-infected cells can be a practical way to increase the transport of antiviral drugs to infected cells to enhance the drug efficacy and reduce undesirable side effects.

Additionally, liposomes containing antiviral agents can be a promising approach for effective virus treatment because they encourage more effective penetration of antiviral agent through the cells, mucus, or skin [93]. The interferon α-2b (IFNα-2b)-loaded PEGylated liposomes were developed for the treatment of HPV vaginal infections because of their mucus-penetrating ability [94]. The IFNα-2b from PEGylated liposomes showed a high penetration ratio through the sheep vaginal tissue compared to free IFNα-2b [94]. The siRNA targeting recombinant open reading frame 7 (r/si-ORF7) of the VZV genome has emerged as a promising approach for RNA interference (RNAi)-based antiviral strategies, offering a novel method for combating VZV [95]. Employing flexible nanoliposomes for the delivery of r/si-ORF7 into both cellular environments and a 3-dimensional (3D) human epidermal skin model—accurately simulating VZV infection in humans—this strategy has been validated. The deployment of r/si-ORF7 encapsulated within nanoliposomes has demonstrated significant success in suppressing VZV replication and substantially reducing viral load in both in vitro and ex vivo settings [95].

Some liposome formulations can increase their immune-evasive ability. For example, liposomes composed of 1-palmitoyl-2-oleoyl-sn-glycero-3-phosphocholine (POPC):1,2-dipalmitoyl-sn-glycero-3-phosphoethanolamine-N-methoxy(polyethylene glycol) (DPPE-PEG2000) (9:1) showed higher immune-evasive ability compared to conventional liposomes of 100% POPC [96]. When the immune-evasive liposome loaded both HIV-1 entry inhibitors [a fusion inhibitor: enfuvirtide (EFV) and an attachment inhibitor: protoporphyrin IX (PPIX)], the dual-loaded liposome enhanced synergy against HIV-1 entry compared to the free forms of drugs [96]. The results may be from the sustainable release of the HIV-1 entry inhibitors at the viral entry site and immune-evasive ability.

Lipid nanoparticles also present several significant challenges that require attention. Concerns regarding potential toxicity arise due to the composition and size of these nanoparticles, which may contribute to adverse effects [97]. Furthermore, the limited drug loading capacity of lipid nanoparticles could diminish therapeutic efficacy, as they can only encapsulate a finite amount of the antiretroviral agent [98]. Stability issues such as aggregation or drug leakage further impact the shelf-life and performance of these nanocarriers [99]. Interactions with the immune system represent another hurdle, as undesired immune responses may reduce the effectiveness of antiretroviral drugs [100]. Lastly, effectively targeting specific cell types or tissues with lipid nanoparticles poses difficulties, potentially restricting their ability to deliver drugs to intended sites of action [101]. Addressing these challenges through continued research and optimization is essential to fully harness the potential of lipid nanoparticles in antiretroviral treatments.

### Polymeric nanoparticle

Polymeric nanocarriers have garnered significant attention as an antiviral agent delivery system because of their adaptable structures, allowing for maximum encapsulation and targeted delivery of agents to specific locations or in response to diverse physiological or external stimuli, improved bioavailability, and retention time [60,102]. Polymeric nanocarriers include various types such as polymeric nanoparticles, polymeric micelle, dendrimer, polymeric hydrogel, and carbon-based nanomaterials.

Biodegradable polymer has been utilized as a multi-antiviral agent-loaded polymeric delivery system to virus-infected cells (Table 3). For example, PLGA has been utilized as a multi-antiviral drug delivery system for antiviral treatment. Three antiretroviral drug [elvitegravir, tenofovir alafenamide, and emtricitabine (EVG/TAF/FTC)]-encapsulated PLGA nanoparticles induced steady-state drug release and targeted to HIV-infected cells. The multi-antiviral drug-loaded PLGA delayed drug release and restricted systemic clearance, enabling long-acting drug administration for treating chronic HIV-1 [103]. Other 3 antiviral drug [ritonavir, lopinavir, and efavirenz (RTV/LPV/EFV)]-encapsulated PLGA nanoparticles prolonged release of the drugs for 28 days. Moreover, these particles penetrated macrophages [104]. The EFV and lopinavir/ritonavir (EFV/LPV/r)-loaded PLGA nanoparticles effectively inhibited HIV-1 infection and transduction, resulting from efficient uptake of antiviral drugs in HIV-1-infected human CD4 cells compared to the free form of drugs [105]. Biodegradable nanoparticles made from poly(β-amino ester) (PBAE) were employed to transport HPV16 E7-targeted CRISPR and short hairpin RNA (shRNA) systems, aimed at silencing and knocking out genes, respectively [106]. These systems are designed to navigate through the biological barriers presented by organs, tissues, and cells [106]. By delivering CRISPR/shRNA specifically aimed at HPV16 E7, these polymeric nanoparticles were effective in reducing the expression of this gene, offering a novel approach to tackle HPV infections and the resulting cervical cancers [106]. This strategy showcases the potential of using targeted, biodegradable nanoparticle systems for precision medicine in combating viral infections and their related malignancies. The HIV-1 p24-Nef peptide/FLiC (flagellin molecule sequence from *Pseudomonas aeruginosa*) conjugate was engineered into a nanovaccine using PLGA nanoparticles [107]. This choice was made because PLGA nanoparticles act as stable carriers for antigens, significantly enhancing the induction of the immune response. Consequently, mice vaccinated with the HIV-p24-Nef/FLiC/PLGA formulation not only demonstrated improved vaccine immunogenicity but also required a lower immunogenic dose of the vaccine candidate [107].

**Table 3.** Example of polymeric nanoparticles for antiviral therapy. This table summarizes various polymeric nanoparticles, encapsulated agents they carry, their target viruses, and key properties.

Polymeric nanoparticle				
Nanoparticle	Encapsulated agents	Targeted virus	Properties of nanoparticles	Ref.
PLGA	Elvitegravir, tenofovir alafenamide, and emtricitabine (EVG/TAF/FTC)	HIV	Multi-antiviral drug delivery long-acting drug administration	[106]
PLGA	Ritonavir, lopinavir, and efavirenz (RTV/LPV/EFV)	HIV	Multi-antiviral drug delivery long-acting drug administration penetrating macrophages	[107]
PLGA	Efavirenz and lopinavir/ritonavir (EFV/LPV/r)	HIV	Multi-antiviral drug delivery long-acting drug administration targeting to HIV-1-infected human CD4 cells	[108]
Nanoparticle made from poly(β-amino ester) (PBAE)	CRISPR/shRNA	HPV	Gene-editing agent delivery Passing through the biological barriers presented by organs, tissues, and cells	[109]
PLGA	HIV-1 p24-Nef/FLiC conjugate	HIV	Vaccine delivery Stable antigen carrier	[110]
PLGA containing CCR5-targeting molecules	Peptide nucleic acids (PNAs) and donor DNAs	HIV	Gene-editing agent delivery Modification of CCR5 gene in human immune cells resistance to HIV-1	[111]
Cationic polymeric nanoparticles, composed of CX3CL1-PEG45-polycaprolactone (PCL)20-saccharide-(COOH)n block copolymer.	siRNA/scRNA	CMV	Gene-editing agent delivery Targeting human CMV-infected cells	[112]
Mannosylated dendrimer	Lamivudine (3TC)	HIV	Antiviral drug delivery targeting HIV-infected macrophage	[114]
Tuftsin-decorated dendrimer	Efavirenz (EFV)	HIV	Antiviral drug delivery attached to mononuclear phagocytic cells improved their natural killer function	[115]
Poly(ε-caprolactone) nanocarrier incorporated into chitosan	Imiquimod	HPV	Antiviral drug delivery muco-adhesion penetration to vaginal tissue drug retention time	[116]
Glutaraldehyde-crosslinked chitosan nanoparticles	Foscarnet (FCN)	CMV	Antiviral drug delivery Acing as an ionotropic agent Controlling drug release	[117]

Polymeric nanoparticles modified with targeting molecules have demonstrated their efficacy in specifically targeting viral diseases for treatment. These modifications enable the nanoparticles to recognize and bind to specific cellular receptors or proteins associated with viral infections, thereby delivering therapeutic agents directly to the infected cells. This targeted approach enhances the effectiveness of antiviral treatments by focusing the therapeutic action on the site of infection, minimizing side effects, and improving patient outcomes. The utilization of nanoparticles for gene-editing therapy offers a significant advantage in antiviral treatment due to their precise targeting capabilities and minimal cytotoxicity. For instance, PLGA containing CCR5-targeting agents demonstrated effective delivery of encapsulated peptide nucleic acids (PNAs) and donor DNAs to modify the CCR5 gene in human peripheral blood mononuclear cells (PBMCs), showing minimal off-target effects on the CCR2 gene [108]. Mice treated with these modified PBMCs exhibited significant resistance to HIV-1, evidenced by higher CD4^+^ T cell counts and lower viral RNA levels [108]. This nanoparticle represents the permanent inactivation of the CCR5 receptor in T cells of HIV-1-infected individuals, suggesting a potential cure for HIV-1 infections. To specifically target HCMV-infected cells, a CX_3_CL_1_ ligand, targeting the HCMV-encoded CX_3_CL_1_ chemokine receptor expressed on these cells, was attached to the surface of the cationic polymeric nanoparticles, composed of CX_3_CL_1_-PEG_45_-polycaprolactone (PCL)_20_-saccharide-(COOH)_n_ block copolymer [109]. CX_3_CL_1_-conjugated polymeric nanoparticles were utilized to deliver small interfering RNA (siRNA) and small complementary RNA (scRNA), thereby inhibiting 2 critical HCMV genes, IE1 and IE2. This strategic modification facilitated the targeted delivery of siRNA/scRNA to the HCMV-infected cells, effectively inhibiting HCMV replication both in vitro and in vivo [109]. Dendrimers, known for their unique structural characteristics such as uniform size distribution, compact globular shapes, high peripheral functional group density, and customizable surface functionalities, have been further modified to enhance their targeting capabilities. By tailoring the surface properties of dendrimers, researchers can significantly increase their affinity for specific viral components or infected cells, thereby optimizing the delivery of therapeutic agents and increasing the efficacy of antiviral treatments [110]. Antiretroviral drug [lamivudine (3TC)]-loaded dendrimers were surface-modified with mannose to target HIV-infected macrophages [111]. The drug delivery system efficiently encapsulated 3TC and gradually released the drug over 144 h. When the dendrimers were surface-modified with mannose for HIV-infected macrophage targeting ability, cellular uptake of 3TC was 21 times higher than the free 3TC. Therefore, the 3TC-loaded mannosylated-dendrimer nanocarrier had stronger anti-HIV effectiveness at concentrations as low as 0.019 nM [111]. Dendrimers chemically conjugated with tuftsin exclusively attached to mononuclear phagocytic cells and improved their natural killer function [112]. When EFV-loaded, tuftsin-conjugated dendrimers were tested in vitro for targeting potential to HIV-infected macrophages, they showed 34.5 times higher cellular uptake than those of the free drug [112]. These findings showed that ligand-conjugated dendrimers had the potential to improve anti-HIV efficacy while lowering the toxicity of antiviral drugs. Therefore, dendrimers with anti-viral drugs would be a good strategy for latency virus disease therapies.

The nanocarrier can be incorporated into the hydrogel to enhance muco-adhesion and penetration of agents for the treatment of infectious diseases. For example, poly(ε-caprolactone) nanocarrier, which loaded imiquimod, was incorporated into chitosan to improve the drug performance for HPV treatment, resulting from enhancing the imiquimod contact to the vaginal tissue, drug retention time, and permeation [113]. Glutaraldehyde-crosslinked chitosan nanoparticles for foscarnet (FCN) delivery demonstrated in vitro antiviral activity against HCMV-infected lung fibroblast cells. The FCN-loaded chitosan nanoparticles, acting as an ionotropic agent, improved the antiviral efficacy by controlling drug release from the nanoparticles [114].

Polymeric nanoparticles encounter several significant challenges in their application for HIV treatment. Potential toxicity concerns stem from the inherent properties of the polymers used, which may lead to adverse effects depending on their composition and size [115]. The limited drug loading capacity of polymeric nanoparticles can diminish therapeutic efficacy, as they can only encapsulate a finite amount of antiretroviral agents [116]. Stability issues such as aggregation or drug leakage further affect the shelf-life and performance of these nanocarriers. Interactions with the immune system present another obstacle, as undesired immune responses may decrease the effectiveness of antiretroviral drugs [60]. Effectively targeting specific cell types or tissues with polymeric nanoparticles also proves challenging, potentially restricting their ability to deliver drugs to intended sites of action in the body. Furthermore, the absence of FDA-approved polymeric nanoparticle formulations for HIV treatment underscores the necessity for additional research and optimization to address these limitations and fully exploit their potential in antiretroviral therapy [117].

### Inorganic nanoparticles

Inorganic nanoparticles, derived from both metallic and nonmetallic sources, have been explored as agents for delivery systems. Their small size facilitates easy entry into cells and tissues, minimizing host immune responses due to their inert nature. Their modifiable size and shape, high surface area, ease of functionalization, crystallinity, and the ability for high-density attachment of surface ligands lead to inorganic nanoparticles being utilized as nanocarriers. Moreover, these inorganic particles exhibit distinctive optical, thermodynamic, magnetic, catalytic, and electrochemical properties, accompanied by supplementary bioactivities [118]. Therefore, inorganic nanoparticles prove to be valuable for clinical applications in antiviral therapy (Table 4).

**Table 4.** Example of inorganic nanoparticles for antiviral therapy. This table summarizes various inorganic nanoparticles, encapsulated agents they carry, their target viruses, and key properties.

Inorganic nanoparticle				
Nanoparticle	Encapsulated agents	Targeted virus	Properties of nanoparticles	Ref.
Mercaptoethane sulfonate (MES) capped silver nanoparticles	-	HSV	Antiviral drug delivery Targeting ability to the HSV-1 virus Competing with HSV for binding to cellular heparan sulfate Blocking viral entry into the cell	[123]
Gold nanoparticles	Gallic acid	HSV	Antiviral drug delivery Antioxidant activity Inhibition of viral attachment Inhibition of viral penetration into Vero cells	[117]
Magneto-electric nanoparticles (MENPs)	Cas9/gRNA	HIV	Gene-editing agent delivery Magnetically guided delivery Noninvasive CNS delivery On-demand Cas9/gRNA release	[124]
Gold nanoparticles	Raltegravir	HIV	Antiviral drug delivery PBMC and macrophage-targeting ability easy entry into cells and tissues	[125]
Inorganic–organic hybrid nanoparticles (IOH-NPs), consisting of [ZrO]^2+^[(FCN)_0.4_(OH)_0.8_]^2−^ and Gd^3+^[FCN]^3−^	Foscarnet (FCN)	CMV	Antiviral drug delivery Viral DNA polymerase inhibition Carrying high drug amounts Low material complexity	[126]
HSV-2 glycoprotein and CAP adjuvant combination	-	HSV	Mucosal vaccine adjuvant Systemic and mucosal immunity against the HSV virus No side effects observed, and there was no induction of IgE antibodies	[129]

Researchers augment the targeting precision of inorganic nanoparticles by attaching surface ligands or utilizing their inherent properties, like magnetic guidance. This approach improves the nanoparticles’ ability to selectively navigate to specific sites within the body, facilitating targeted therapeutic interventions. Silver nanoparticles (AgNPs) capped with mercaptoethane sulfonate (MES) were designed to target the HSV-1 virus and compete for their binding to cellular heparan sulfate through their sulfonate end groups. MES-capped AgNPs showed effective inhibition of HSC-1 infection in vitro, resulting from blocking viral entry into the cell to prevent the following infection [119]. The attachment of Cas9/gRNA to magneto-electric nanoparticles (CRISPR/Cas9-gRNA-MENPs) allows for their magnetic navigation across the blood–brain barrier (BBB) in a noninvasive manner, targeting the reduction of latent HIV-1 within microglial (hμglia)/HIV (HC69) cells [120]. This technique improves the cellular uptake of the CRISPR/Cas9-gRNA-MENPs via nanoelectroporation and ensures the targeted release of Cas9/gRNA (98%) through the activation of an alternating magnetic field [120]. This activation promotes the separation of Cas9/gRNA from MENPs’ surfaces, resulting in its release inside the cells and the effective suppression of HIV.

Moreover, inorganic nanoparticles enable the attachment of various drugs to their surfaces, allowing for the simultaneous transport of multiple therapeutic agents due to their multivalent nature. This capability enhances the efficiency of drug delivery systems by enabling the coordinated delivery of different treatments within a single nanoparticle platform. For instance, gold nanoparticles were linked with raltegravir molecules, recognized for their ability to inhibit HIV, and were tested in infected primary PBMCs that were depleted of CD8^+^ cells. The delivery of raltegravir-conjugated AuNPs demonstrated antiviral activity in HIV-infected primary lymphocytes because of their PBMC and macrophage-targeting ability [121]. AuNPs conjugated with gallic acid, known for its antioxidant activity, demonstrated antiviral efficacy against HSV infections in Vero cells. AuNPs conjugated with gallic acid, known for its antioxidant activity, demonstrated antiviral efficacy against HSV infections in Vero cells. Gallic acid-conjugated gold nanoparticles (AuNPs) effectively prevented viral attachment and penetration into the cells, demonstrating no significant cytotoxic effects [122]. FCN, a viral DNA polymerase inhibitor, was capped on inorganic–organic hybrid nanoparticles (IOH-NPs), consisting of [ZrO]^2+^[(FCN)_0.4_(OH)_0.8_]^2−^ and Gd^3+^[FCN]^3−^. These FCN-capped IOH-NPs demonstrated antiviral activity against HCMV, characterized by their ability to carry high drug amounts and low material complexity [123].

Sometimes, inorganic nanoparticles play a crucial role as antiviral agents on their own. Mice vaccinated with an HSV-2 glycoprotein and calcium phosphate nanoparticle (CAP) adjuvant combination (HSV-2 + CAP) developed HSV-specific immunoglobulin A (IgA) and IgG antibodies at mucosal sites while also showing an increase in systemic IgG responses [124]. This dual action indicates a robust enhancement of both systemic and mucosal immunity against the live virus, achieved through immunization with the CAP-based HSV-2 subunit vaccine [124]. Mice receiving the HSV-2 + CAP vaccine demonstrated protection against live HSV-2 infection. The absence of side effects and the lack of IgE antibody induction with CAP highlight its potential as a safe and effective mucosal vaccine adjuvant for human use [124].

Inorganic nanoparticles present several potential disadvantages when applied to HIV patients. They may inadvertently impact the compromised immune system of HIV patients by inducing unwanted immune activation, which can lead to heightened inflammation and unintended effects off-target [125]. Conversely, certain nanoparticles might possess immunosuppressive properties, further compromising the already weakened immune system in HIV patients. Moreover, inorganic nanoparticles have the potential to interfere with the pharmacokinetics or effectiveness of antiretroviral drugs, potentially undermining HIV treatment outcomes and immune recovery [126]. Theoretically, nanoparticles could even facilitate HIV entry into target cells or promote viral replication, exacerbating the underlying immune dysfunction. These complex interactions between inorganic nanoparticles and the immune system affected by HIV underscore the necessity for meticulous evaluation and optimization to ensure the safe and effective utilization of these nanomaterials in HIV treatment. Addressing these potential disadvantages is essential for fully realizing the therapeutic benefits of inorganic nanoparticles for HIV patients.

## Future Perspective

In the realm of future therapies, nanoparticle-based antiretroviral treatments hold promise for surmounting current limitations. Polymeric nanoparticles will leverage biocompatible and biodegradable polymers such as PLGA, chitosan, or gelatin to mitigate potential toxicity concerns. Enhanced drug loading capacities will be achieved through refined polymer compositions and nanoparticle formulations, while surface modifications promise improved stability. Targeted delivery mechanisms will be realized through the strategic conjugation of nanoparticles with ligands or antibodies that bind specifically to receptors on HIV-infected cells. Lipid nanoparticles will be meticulously engineered with tailored lipid compositions to enhance biocompatibility and reduce toxicity risks. Advanced encapsulation techniques and the use of lipids with superior drug solubility will further optimize drug loading capacities. Stability will be bolstered through the incorporation of stabilizers like cholesterol or PEGylation, complemented by targeted delivery facilitated by surface modifications employing ligands or antibodies designed for HIV-infected cells.

Inorganic nanoparticles, carefully designed with consideration for composition and surface properties, will aim to minimize potential toxicity and interactions with the immune system. Strategies including porous structures and drug incorporation into the nanoparticle matrix will enhance drug loading capacities. Targeted delivery will be achieved through conjugation with ligands or antibodies specific to HIV-infected cells. Research will additionally explore the impact of inorganic nanoparticles on HIV replication and latency, providing critical insights into their effects on the immune system. Comprehensive evaluation through rigorous in vitro and in vivo studies will assess the safety, efficacy, and targeted delivery capabilities of these innovative nanocarriers. Collaboration between experts in nanomedicine and HIV/acquired immunodeficiency syndrome (AIDS) research will be essential for identifying the most promising nanoparticle strategies and overcoming inherent system limitations. Ultimately, the development of nanoparticle-based antiretroviral therapy holds great potential to revolutionize HIV treatment by offering more effective, targeted options, potentially leading to improved treatment outcomes and enhanced quality of life for patients.

## Conclusion

In summary, we underscore the intricate nature of viral latency and its critical role in the survival and persistence of viruses such as herpesviruses, HIV, and HPV within the host. The exploration of episomal and proviral latency mechanisms reveals the complex strategies viruses employ to evade eradication by integrating into or existing alongside the host’s genetic material. This understanding is pivotal for addressing the significant challenges in eradicating latent reservoirs, particularly in HIV, where conventional therapies have fallen short. We highlight the innovative potential of nanotechnology, specifically through the use of nanoparticle-based drug delivery systems, gene-editing techniques, and vaccines, as a transformative approach in antiviral therapy. The targeted, efficient, and sustained delivery capabilities of nanoparticles, combined with the promise of epigenetic and genetic interventions, offer novel therapeutic avenues to disrupt viral latency and activate the immune response against latent viruses. The integration of nanotechnology with a deep molecular understanding of viral latency opens new horizons for therapeutic interventions, potentially revolutionizing the management of persistent viral infections and heralding a new era in the fight against diseases like neuroHIV/AIDS, herpes, and HPV. We ultimately call for further investigation and validation of these nanotechnological approaches to fully harness their potential in clinical applications, paving the way for groundbreaking advancements in antiviral therapy.

## Data Availability

No data were used for the research described in the article.
